# Ensiling characteristics and in vitro rumen fermentation of *Sesbania grandiflora* pods prepared with inhibitory and stimulatory additives

**DOI:** 10.1038/s41598-025-05095-w

**Published:** 2025-07-02

**Authors:** Chatchai Kaewpila, Pongsatorn Gunun, Pongsathorn Tongkasee, Premsak Puangploy, Waroon Khota

**Affiliations:** 1 Faculty of Natural Resources, Rajamangala University of Technology Isan, Sakon Nakhon Campus, Sakon Nakhon, 47160 Thailand; 2 Faculty of Digital Agro-Industry, King Mongkut’s University of Technology North Bangkok, Prachinburi Campus, Prachinburi, 25230 Thailand

**Keywords:** Biotechnology, Zoology

## Abstract

Limited research has been conducted on the ensiling characteristics of *Sesbania grandiflora* pods (SGP) for ruminant production. The main objective of this study was to evaluate the effects of silage additives on ensiling characteristics and in vitro rumen fermentation of SGP. A completely randomized design included three treatments: control (untreated), formic acid (FA) at 7.5 mL/kg fresh matter (FM) as an inhibitor, and sugarcane molasses at 50 g/kg FM with *Lactobacillus casei* TH14 at 1.0 × 10^5^ colony forming units/g FM (MO + TH14) as a stimulator. The ensiling process lasted 30 days. SGP material showed high levels of soluble true protein and polyunsaturated fatty acids. However, it also exhibited low in vitro dry matter digestibility (IVDMD) and the presence of bis(2-ethylhexyl) phthalate, a critical toxic compound. Compared to the control, both additives reduced ensiling loss, pH (≤ 3.82), butyric acid, and ammonia nitrogen levels. Compared to the other treatments, the MO + TH14 treatment showed greater concentrations of total volatile fatty acid (VFA) and propionic acid, while reducing in vitro CH_4_ emission intensities expressed as g/kg IVDMD and mol/mol total VFAs. In conclusion, the results highlight the nutritive value and phytochemical composition of SGP, demonstrating the significant impact of MO + TH14 as a stimulatory additive in enhancing lactic acid fermentation, improving nutritive value, and reducing in vitro CH_4_ emission intensities in SGP silage. The detection of bis(2-ethylhexyl) phthalate in SGP represents a significant toxicological concern with potential implications for food and feed safety, warranting further investigation into its source and associated risks. Further research is essential to test SGP silage in vivo to ensure its benefits and limitations in ruminant production.

## Introduction

To meet population demands, ruminant producers worldwide face significant challenges due to feed shortages and high enteric methane (CH_4_) emissions^1,2^. Recently, in tropical countries, nutrient- and phytochemical-rich indigenous plants have been recommended as a promising strategy to enhance ruminant productivity and health while reducing enteric CH_4_ emissions^3,4^. However, determining the appropriate use of these plants, whether as feedstuffs, feed additives, or a combination of both, remains a challenge in terms of practical application.

*Sesbania grandiflora* (L.) Poir is a small, erect, fast-growing, and sparsely branched tree that thrives primarily in hot and humid environments worldwide, particularly in India and Southeast Asia^5,6^. This versatile plant is highly valued for its edible and medicinal parts^7 ^with flowers and young pods commonly consumed as vegetables^8 ^leaves used as feed for livestock^9 ^and mature pods employed as a phytogenic feed additive for ruminants^10^. The seeds are reported to be a source of plant metabolites, including alkaloids, flavonoids, saponins, phenols, tannins, and others, which exhibit antibacterial effects^11^. Unnawong et al.^10^ demonstrated that supplementing beef cattle diets with whole *S. grandiflora* pod (SGP) meal at a rate of 6 g/kg dry matter (DM) can increase ruminal concentrations of total volatile fatty acids (VFAs) and propionic acid, while simultaneously reducing enteric CH_4_ emissions and fecal nitrogen (N) excretion. Interestingly, SGP also contains a high crude protein (CP) content of 217.3 g/kg DM. Currently, no data reveals the use of SGP as a nutrient source. Ngwa et al.^12^ showed that ensiled legume tree pods had higher feed intake and palatability among sheep and goats compared to their dried form. Therefore, ensiled SGP may also have favorable feeding characteristics for ruminants, despite its high level of phytochemical compounds.

Ensiling is a widely recognized method for preserving the nutritive value of high-moisture plant materials, enabling year-round feeding for ruminants^13,14^. Improving ensiling characteristics is essential for effective production and utilization of silage in animals. Key factors in pre-ensiling materials, such as optimal moisture content, low lactate-buffering capacity (LBC), sufficient water-soluble carbohydrates (WSC), and an adequate population of lactic acid bacteria (LAB), play a crucial role in the fermentation process^14,15^. When these natural factors are insufficient, the use of optimal silage additives, including inhibitors and stimulators, becomes necessary^14^. These additives can effectively modify the biochemical processes in silage, improving its quality and utilization in ruminant feeding^15^. Wei et al.^16^ demonstrated that formic acid (FA) at 5.0 mL/kg fresh matter (FM) can inhibit silage fermentation, while LAB inoculation at approximately 10^6^ colony forming units (cfu)/g FM promotes it. Among homofermentative LAB strains, *Lactobacillus casei* TH14 has demonstrated specific advantages in tropical forages by increasing lactic acid production relative to other fermentation products, reducing pH, and enhancing the chemical composition of silage^17^. Molasses, a commonly used feedstuff in tropical regions, provides fermentable WSC that support the activity of LAB during the ensiling process^18^. Wang et al.^19^ found that adding molasses at 25 to 50 g/kg FM enhances the fermentation quality of mixed silage. However, there are reports stating additive effectiveness varies with crop type^16^. Moreover, the addition of optimal silage additives may offer new strategies for mitigating enteric CH_4_ emissions^20^. Thus, evaluating additive suitability for SGP silage is essential to identify practical and sustainable options.

In this context, SGP has attracted interest as a dual-purpose plant, offering both nutritional value and phytogenic compounds with potential for CH_4_ mitigation in tropical ruminant systems. However, despite these promising attributes, SGP has not been extensively studied as a primary silage material, and its ensiling potential and post-ensiling rumen fermentation characteristics in response to various silage additives remain largely unknown. These knowledge gaps underscore the importance of investigating silage additives as a strategy to improve the utilization of SGP and further develop its potential as a CH_4_ mitigation resource. We hypothesize that additives could play an essential role in enhancing the nutrient availability of SGP silage. Therefore, the main objective of this study was to investigate the effects of silage additives on ensiling end-products, microbial counts, ensiling loss, aerobic stability, chemical composition, and in vitro rumen fermentation of SGP silage.

## Results

### Nutritional, microbial, and phytochemical profiles of SGP

The chemical composition, in vitro dry matter digestibility (IVDMD), pH, LBC, and microbial counts of SGP are presented in Table 1. The DM content was recorded at 208 g/kg. The organic matter (OM), CP, and ether extract (EE) contents were 919, 221, and 24.5 g/kg DM, respectively. The neutral detergent fiber (NDF), acid detergent fiber (ADF), and acid detergent lignin (ADL) were high, ranging from 158 to 533 g/kg DM. The WSC content was 29.9 g/kg DM. The protein fractions per kg DM included 30.3 g soluble true protein, 83.7 g soluble non-true protein, and 107 g insoluble protein. The IVDMD was remarkably low at 365 g/kg. The pH was 5.97, with an extremely high LBC of 1,175 milliequivalents (mEq)/kg DM. Microbial populations ranged from 4.23 to 5.40 log10 cfu/g FM, predominantly consisting of yeasts and aerobic bacteria.

**Table 1:** Chemical composition, IVDMD, pH, LBC, and microbial counts of SGP material used in this study.

Item	Value
**Chemical composition (g/kg DM)**	
DM (g/kg)	207.87
OM	919.37
CP	220.68
EE	24.49
NDF	533.36
ADF	437.22
ADL	157.65
WSC	29.87
Soluble true protein	30.34
Soluble non-true protein	83.65
Insoluble protein	106.69
IVDMD (g/kg)	365.32
pH	5.97
LBC (mEq/kg DM)	1,175.14
**Microbial counts (log10 cfu/g FM)**	
LAB	4.67
Aerobic bacteria	5.11
Coliform bacteria	4.88
Yeasts	5.40
Molds	4.23

*IVDMD* in vitro dry matter digestibility (after 24 h of incubation), *LBC* lactate-buffering capacity, *SGP S. grandiflora* pods, *DM* dry matter, *OM* organic matter, *CP* crude protein, *EE* ether extract, *NDF* neutral detergent fiber, *ADF* acid detergent fiber, *ADL* acid detergent lignin, *WSC* water-soluble carbohydrates, *Eq* equivalents; *cfu* colony-forming units, *FM* fresh matter, *LAB* lactic acid bacteria.

The lipid content of SGP was rich in unsaturated fatty acids, which accounted for two-thirds of total fatty acids (Table 2). Polyunsaturated fatty acids (PUFAs) were recorded at 563 g/kg of the total. The predominant PUFAs included linolelaidic and linoleic acids (478 g/kg total), α-linolenic acid (73.0 g/kg total), and docosadienoic acid (9.17 g/kg total).

**Table 2:** Fatty acid profile of SGP material used in this study.

Fatty acid profile (g/kg total fatty acids)	Value
C4:0 − Butyric acid	2.68
C6:0 − Caproic acid	3.06
C8:0 − Caprylic acid	2.68
C10:0 − Decanoic acid	1.53
C14:0 − Myristic acid	4.20
C14:1 − Myristoleic acid (cis-9)	2.68
C15:0 − Pentadecanoic acid	2.29
C15:1 − Pentadecenoic acid (cis-10)	2.29
C16:0 − Palmitic acid	212.92
C16:1 − Palmitoleic acid	2.68
C17:0 − Heptadecanoic acid	4.59
C18:0 − Stearic acid	73.39
C18:1 − Octadecenoic acid (trans-9) and oleic acid (cis-9)	98.24
C18:2 − Linolelaidic acid (trans-9,12) and linoleic acid (cis-9,12)	478.21
C18:3 − α-Linolenic acid (n3, cis-9,12,15)	73.01
C20:0 − Behenic acid	9.17
C20:1 − Eicosenoic acid (cis-11)	1.53
C20:2 − Eicosadienoic acid (cis-11,14)	2.29
C22:0 − Behenic acid	4.59
C22:1 − Erucic acid (cis-13)	1.53
C22:2 − Docosadienoic acid (cis-13,16)	9.17
C23:0 − Tricosanoic acid	3.06
C24:0 − Lignoceric acid	4.20
Saturated fatty acids (SFAs)	328.36
Monounsaturated fatty acids (MUFAs)	108.94
Polyunsaturated fatty acids (PUFAs)	562.69

*SGP S. grandiflora* pods.

The methanolic extract of SGP was recorded at 45.0 g/kg DM (Table 3). In addition to this, those extracted using water (sonicate method), ethanol (950 mL/L in water, sonicate method), and hexane (Soxhlet apparatus) were 52.0, 27.0, and 3.50 g/kg DM, respectively (data not shown). Total phenolics, total flavonoids, and exhibiting antioxidant activity per g of methanolic extract measured at 95.1 mg of gallic acid Eq, 15.7 mg of quercetin Eq, and 182 mg of trolox Eq, respectively. Reagent screening showed the presence of cardiac glycosides, saponins, tannins, and terpenoids, while alkaloids, anthraquinones, and coumarins were absent in the methanolic extract. However, coumarins were detected in the ethanolic extract (data not shown). Gas chromatograph-mass spectrometer (GC-MS) analysis identified five major organic compounds in the methanolic extract of SGP, listed from the largest to the smallest peak areas, as follows: bis(2-ethylhexyl) phthalate, pentanoic acid, butane, propanedioic acid, and dimethylamine.

**Table 3:** Phytochemical composition and GC-MS analysis of SGP extract using methanol as the solvent.

Item	Value
Methanolic extract (g/kg DM)	45.00
**Reagent tests**	
Total phenolic acids (mg gallic acid Eq/g extract)	95.08
Total flavonoids (mg quercetin Eq/g extract)	15.67
Antioxidant activity (mg trolox Eq/g extract)	182.01
Alkaloid (Wagner’s test)	‒
Anthraquinone (Borntrager’s test)	‒
Cardiac glycoside (Keller-Kiliani’s test)	+
Coumarin (NaOH test)	‒
Saponin (frothing test)	+
Tannin (ferric chloride test)	+
Terpenoid (Salkowski’s test)	+
**GC-MS analysis (% of total peak areas)**	
Bis(2-ethylhexyl) phthalate	17.24
Pentanoic acid	4.63
Butane	4.44
Propanedioic acid	1.90
Dimethylamine	1.54

*GC-MS* gas chromatograph-mass spectrometer, *SGP S. grandiflora* pods, *DM* dry matter, *Eq* equivalents, *NaOH* sodium hydroxide, ‒ negative test, + positive test.

### Ensiling end-products of SGP silage

Table 4 presents the effects of silage additives, including the control (untreated), FA as an inhibitor, and a combination of sugarcane molasses with a locally selected *L. casei* TH14 inoculant (MO + TH14) as a stimulator on the ensiling end-products of SGP silage. SGP silage treated with FA and MO + TH14 exhibited significantly lower (p *<* 0.001) pH, propionic acid, butyric acid, and ammonia-N levels. The FA treatment resulted in lower (p *<* 0.001) ethanol and acetic acid concentrations compared to MO + TH14 and the control, respectively. The MO + TH14 treatment showed higher (p *<* 0.001) lactic acid content, while the FA-treated silage had the lowest.

**Table 4:** Ensiling end-products of SGP silage prepared with additives after 30 days of ensiling.

Item	Control	FA	MO + TH14	SEM	*P*-value
pH	5.60^a^	3.82^b^	3.79^b^	0.037	< 0.001
Ethanol (g/kg DM)	27.27^a^	1.92^c^	14.37^b^	0.723	< 0.001
Lactic acid (g/kg DM)	16.68^b^	7.33^c^	69.35^a^	1.169	< 0.001
Acetic acid (g/kg DM)	97.75^a^	4.31^c^	18.07^b^	2.624	< 0.001
Propionic acid (g/kg DM)	68.18^a^	nd^b^	nd^b^	3.276	< 0.001
Butyric acid (g/kg DM)	75.80^a^	0.06^b^	nd^b^	2.612	< 0.001
Ammonia-N (g/kg DM)	5.18^a^	0.35^b^	0.36^b^	0.234	< 0.001

*SGP S. grandiflora* pods, *Control* untreated, *FA* formic acid, *MO* + *TH14* combination of molasses and *L. casei* TH14 inoculant, *SEM* standard error of the means, *DM* dry matter, *N* nitrogen, *nd* not detected. Means within rows with different superscript letters differ at p *<* 0.05.

### Microbial counts, ensiling loss, and aerobic stability of SGP silage

**Fig. 1:**
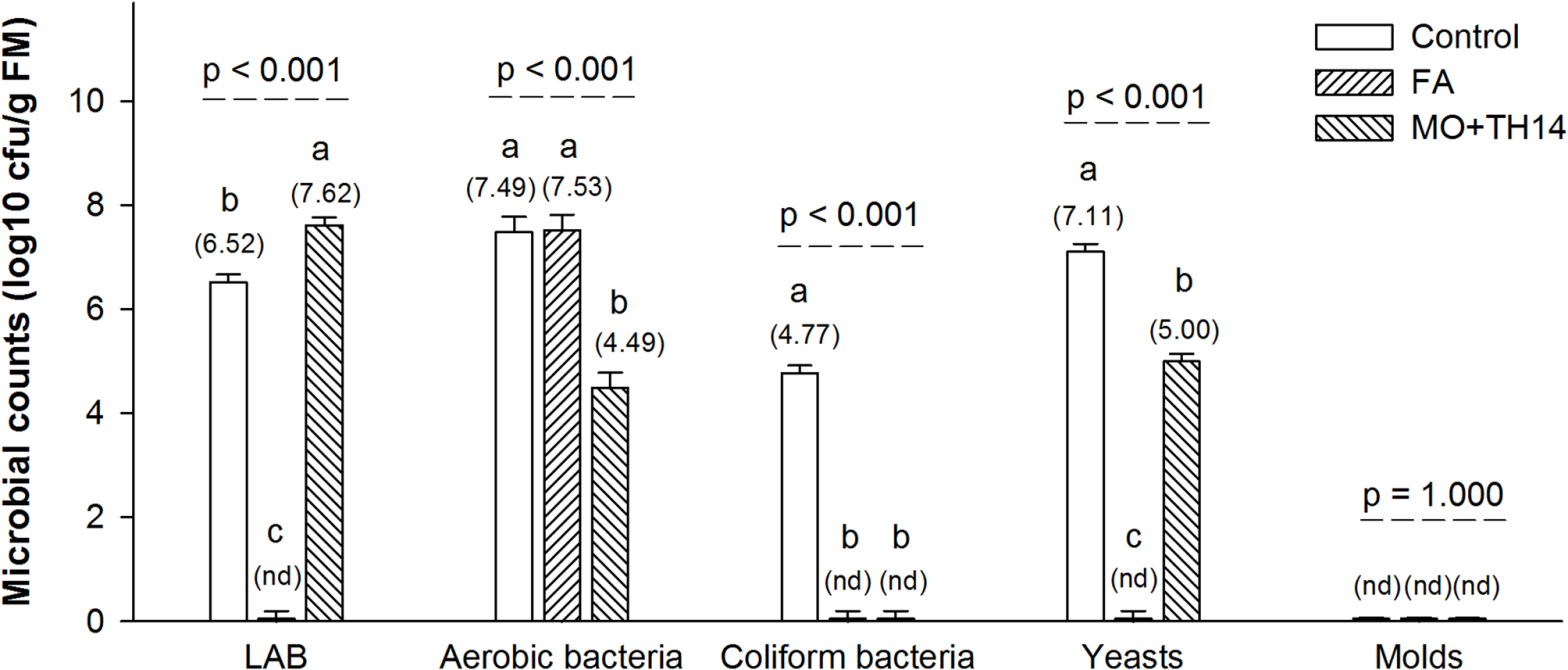
Microbial counts of SGP silage prepared with additives after 30 days of ensiling. *SGP S. grandiflora* pods, *LAB* lactic acid bacteria, *cfu* colony forming units, *FM* fresh matter, *Control* untreated, *FA* formic acid, *MO* + *TH14* combination of molasses and *L. casei* TH14 inoculant, *nd* not detected. Error bars represent the standard error of the means. Means with different superscript letters differ at p *<* 0.05.

The populations of LAB, coliform bacteria, and yeasts in FA-treated silage were below detectable levels (Fig. 1). The MO + TH14 treatment resulted in significantly higher (p *<* 0.001) LAB numbers compared to the control, while aerobic bacteria counts were higher (p *<* 0.001) in control and FA treatments. The control had greater (p *<* 0.001) counts of coliform bacteria and yeasts than the other treatments. Mold counts were below detectable levels across all treatments.

**Fig. 2:**
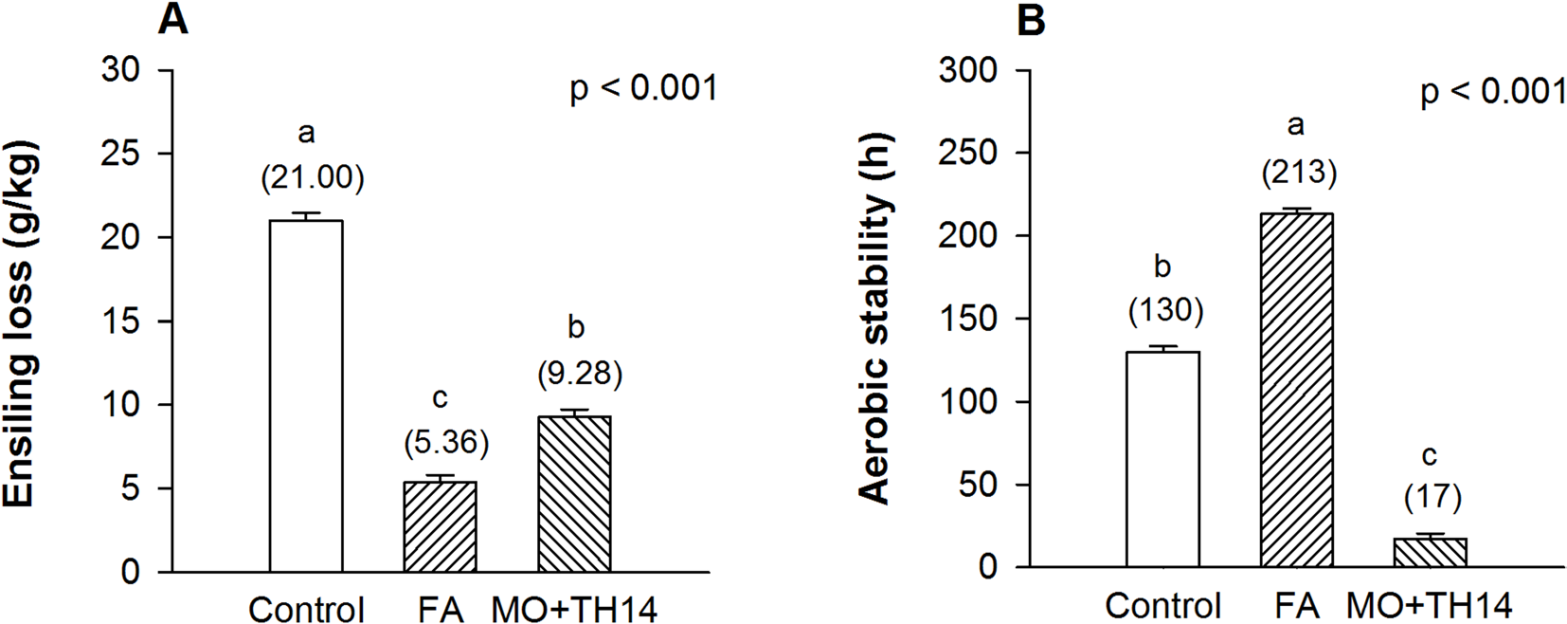
Ensiling loss (**A**) and aerobic stability (**B**) of SGP silage prepared with additives after 30 days of ensiling. *SGP S. grandiflora* pods, *Control* untreated, *FA* formic acid, *MO* + *TH14* combination of molasses and *L. casei* TH14 inoculant. Error bars represent the standard error of the means. Means with different superscript letters differ at p *<* 0.05.

Ensiling loss of the control was recorded at 21.0 g/kg and reduced (p *<* 0.001) with the additives (Fig. 2A). Compared to the control, the aerobic stability period was extended (p *<* 0.001) with FA (130 h vs. 213 h) but reduced with MO + TH14 (17 h) treatment (Fig. 2B).

### Chemical composition of SGP silage

The MO + TH14 treatment resulted in significantly higher (p *<* 0.001) DM content, followed by the FA and control treatments, respectively (Table 5). OM content was higher (p *<* 0.001) in the FA-treated silage compared to the MO + TH14 and control treatments. The FA treatment yielded a higher (p = 0.049) CP content compared to the control, while the MO + TH14 treatment showed intermediate values that did not differ from either the control or FA treatment. EE contents in the control and MO + TH14 treatments were greater (p = 0.005) than in the FA treatment. NDF and ADF contents in the control were greater (p *<* 0.001) than in the FA and MO + TH14 treatments, respectively. ADL content was lower (p = 0.016) in the MO + TH14 treatment compared to the control and FA treatments.

**Table 5:** Chemical composition of SGP silage prepared with additives after 30 days of ensiling.

Item	Control	FA	MO + TH14	SEM	*P*-value
DM (g/kg)	170.65^c^	204.07^b^	219.11^a^	1.064	< 0.001
OM (g/kg DM)	891.12^c^	918.96^a^	910.10^b^	1.970	< 0.001
CP (g/kg DM)	177.81^b^	204.27^a^	186.64^ab^	6.498	0.049
EE (g/kg DM)	51.09^a^	35.93^b^	46.05^a^	2.447	0.005
NDF (g/kg DM)	520.72^a^	466.10^b^	409.42^c^	8.352	< 0.001
ADF (g/kg DM)	411.34^a^	360.38^b^	315.38^c^	10.905	0.001
ADL (g/kg DM)	160.15^a^	158.75^a^	126.36^b^	7.334	0.016

*SGP S. grandiflora* pods, *Control* untreated, *FA* formic acid, *MO* + *TH14* combination of molasses and *L. casei* TH14 inoculant, *SEM* standard error of the means, *DM* dry matter, *OM* organic matter, *CP* crude protein, *EE* ether extract, *NDF* neutral detergent fiber, *ADF* acid detergent fiber, *ADL* acid detergent lignin. Means within rows with different superscript letters differ at p *<* 0.05.

### In vitro rumen fermentation and CH_4_ emissions of SGP silage

Both the FA and MO + TH14 treatments significantly improved (p < 0.001) IVDMD values compared to the untreated silage (Table 6). The levels of total VFAs and propionic acid were higher (p < 0.001) in the MO + TH14 treatment than in the FA and control treatments, respectively. Acetic acid and butyric acid concentrations were greater (p < 0.001) in the FA and MO + TH14 treatments compared to the control. The ratio of propionic acid to acetic acid and the gas production (GP) value in the MO + TH14 treatment were greater (p < 0.001) than those in the other treatments. The value of CH_4_ emissions (g/kg DM) was higher (p < 0.001) in the FA treatment, followed by the MO + TH14 and control treatments, respectively.

**Table 6:** In vitro rumen fermentation parameters after 24 h of incubation of SGP silage prepared with additives following 30 days of ensiling.

Item	Control	FA	MO + TH14	SEM	*P*-value
IVDMD (g/kg)	265.46^b^	400.13^a^	415.14^a^	14.310	< 0.001
VFA concentration (mol/kg DM substrate)					
Total VFAs	1.61^c^	3.27^b^	3.72^a^	0.109	< 0.001
Acetic acid	1.06^b^	2.15^a^	1.98^a^	0.072	< 0.001
Propionic acid	0.43^c^	0.92^b^	1.53^a^	0.036	< 0.001
Butyric acid	0.12^b^	0.20^a^	0.21^a^	0.010	< 0.001
Propionic acid/acetic acid ratio	0.40^b^	0.43^b^	0.78^a^	0.017	< 0.001
GP (L/kg DM)	29.90^c^	65.35^b^	82.69^a^	3.403	< 0.001
CH_4_ emissions (g/kg DM)	6.87^c^	13.36^a^	9.28^b^	0.415	< 0.001

*SGP S. grandiflora* pods, *Control* untreated, *FA* formic acid, *MO* + *TH14* combination of molasses and *L. casei* TH14 inoculant, *SEM* standard error of the means, *IVDMD* in vitro dry matter digestibility, *VFAs* volatile fatty acids, *DM* dry matter, *GP* gas production. Means within rows with different superscript letters differ at p *<* 0.05.

**Fig. 3:**
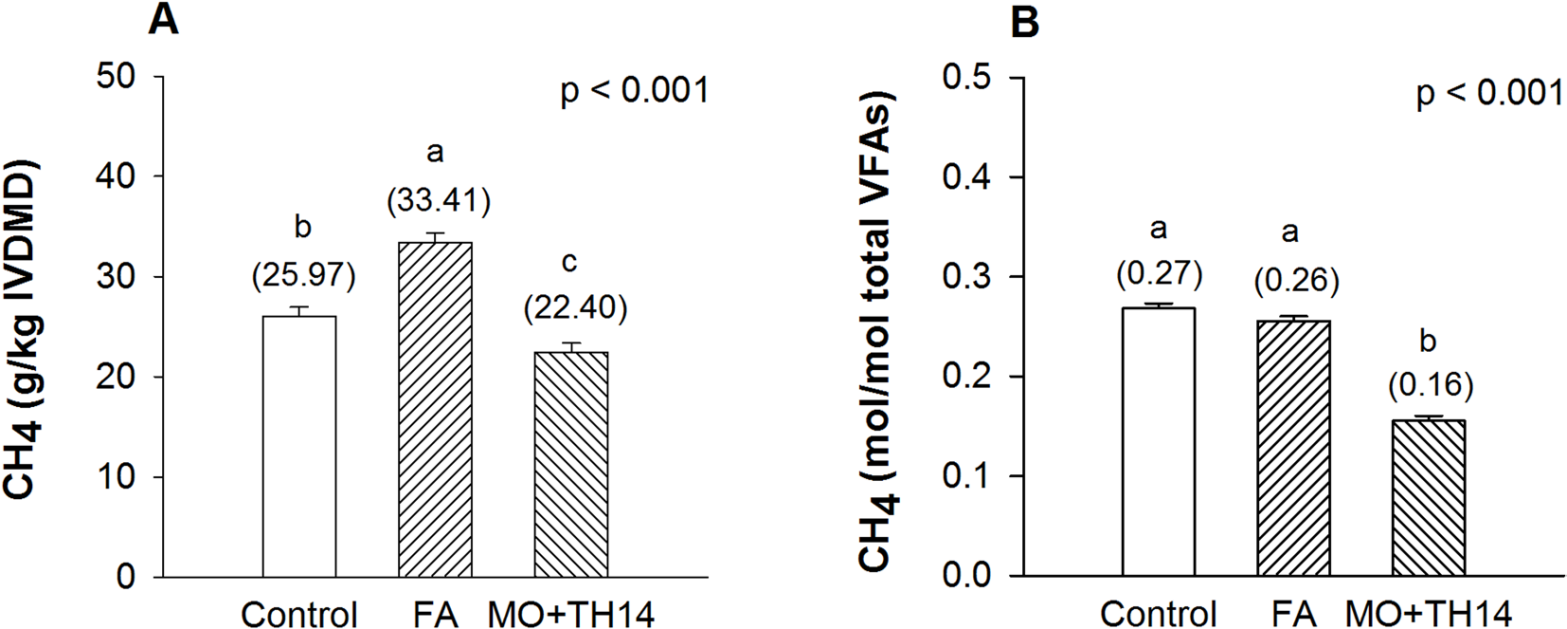
In vitro CH_4_ emission intensities, expressed as g/kg IVDMD (**A**) and mol/mol total VFAs (**B**), after 24 h of incubation of SGP silage prepared with additives following 30 days of ensiling. *CH*_*4*_ methane, *SGP S. grandiflora* pods, *IVDMD* in vitro dry matter digestibility, *VFAs* volatile fatty acids, *Control* untreated, *FA* formic acid, *MO* + *TH14* combination of molasses and *L. casei* TH14 inoculant. Error bars represent the standard error of the means. Means with different superscript letters differ at p *<* 0.05.

CH_4_ emission intensity, expressed as g/kg IVDMD, was lower (p < 0.001) in the MO + TH14 treatment, followed by the control and FA treatments, respectively (Fig. 3A). However, the MO + TH14 treatment exhibited the lowest (p < 0.001) CH_4_ emissions per unit of total VFAs compared to the other treatments (Fig. 3B).

## Discussion

Analyzing the chemical composition and utilization of feedstuffs is essential for assessing their nutritive value in meeting the requirements of ruminants^21^. The results reveal that SGP contains a high level of soluble true protein, with a promising content of 30.3 g/kg DM or 138 g/kg CP (Table 1). This N fraction primarily consists of polypeptides, indicating its biological value (BV)^22^. Compared to soybean meal, which contains approximately 49.9 g of soluble true protein/kg DM^23 ^the soluble true protein content in SGP is 60.8% of that in soybean meal. However, our results also highlight a significant limitation of SGP, as it exhibits a notably low IVDMD value (365 g/kg). This finding can be attributed to the high lignin content in SGP (ADL = 158 g/kg DM), as lignin is known to form lignocellulosic complexes that protect fiber from microbial enzymatic activity^15^. In a study on legume tree pods, Ortiz-Domínguez et al.^24^ reported lignin contents of 82, 159, 243, and 293 g/kg DM in *Caesalpinia gaumeri*,* Acacia pennatula*,* Senegalia gaumeri*, and *Lysiloma latisiliquum*, respectively. The corresponding IVDMD values were 460, 445, 295, and 214 g/kg, indicating a negative relationship between lignin content and digestibility^24^. In addition, our finding of a high LBC in SGP aligns with Ngwa et al.^25^who noted that the pods of *A. sieberiana*, like those of many legumes, can exhibit higher LBC than grasses due to their low sugar content. Ndlovu^26^ further suggested that the high LBC observed in legume species may be associated with their high lignin content. Another possible reason is the high phytochemical composition in SGP, which can exert an antimicrobial effect, thereby decreasing the IVDMD value. A previous study reported a positive relationship between IVDMD and the metabolizable energy content in feedstuffs^27^. Therefore, our findings suggest that the promising energy potential of SGP may be restricted in ruminants. Based on the overall chemical composition and IVDMD values obtained from SGP in this study, the results indicate that it is rich in protein (CP and true protein) and may also contain a substantial amount of indigestible fiber, which aligns with the finding of Unnawong et al.^10^.

PUFAs, including α-linolenic acid and linoleic acid, are essential fatty acids that promote ruminant health^28^ and support the production of functional meat and milk^29^. However, due to biohydrogenation in the rumen, only approximately 100 g/kg of total unsaturated fatty acids escape the rumen and remain available for ruminant utilization^30^. Our findings indicate that SGP can be a potential source of PUFAs, including linolelaidic acid, linoleic acid, α-linolenic acid, docosadienoic acid, and eicosadienoic acid (Table 2). The abundance of these PUFAs in SGP is consistent with the findings of Zain et al.^31^. In the future, oil derived from SGP may offer an additional valuable source of fat, warranting further research. Plant-derived oils, sourced primarily from forage plants and linseed, can be effectively used in ruminant diets^28^. In dairy cows, PUFA supplementation at 20 g/kg DM altered the profile of health-promoting fatty acids in milk^29^. Golbotteh et al.^29^ also stated that PUFA-rich fat supplements can serve as a key source of bioactive and beneficial fatty acids, such as n-3 fatty acids and specific ruminal biohydrogenation intermediates, including conjugated linoleic acid, found in dairy and beef products.

Upon optimization, dietary supplementation with phytogenic feed can offer multiple benefits, including improved rumen fermentation, enhanced health, greater reproductive and productive performance, and increased meat quality^4^. Previous studies have reported that different parts of *S. grandiflora* contain distinct phytochemical composition^32^. Our results indicate that SGP had considerable levels of total phenolics, total flavonoids, and antioxidant properties (Table 3). The presence of flavonoids, saponins, tannins, and terpenoids in the pods was consistent with previous findings in the seeds^11^. Moreover, GC-MS analysis identified five major organic compounds, previously known for their synergistic pharmacological effects as antimicrobial and antioxidant agents^33,34^.

However, it is important to note the detection of bis(2-ethylhexyl) phthalate in SGP (Table 3), a known highly hazardous substance in both humans and animals^35–37^. Shafikova et al.^36^ revealed that phthalates are mutagenic, teratogenic, carcinogenic agents and endocrine disruptors, and have been detected in microorganisms, algae, fungi, and higher plants. Phthalates in plants can originate from plastic waste contamination in soil^35,38^ or may be biosynthesized as part of a defense mechanism against bacterial phytopathogens^36^. This study is among the first to report the presence of bis(2-ethylhexyl) phthalate in SGP, following recent findings in seeds by Rajappan et al.^11^. Presently, phthalates appear to be widespread organic contaminants in farmland soils across agricultural systems^39 ^highlighting the need to understand the current status of phthalate pollution, including their sources, exposure routes, health impacts, and potential remediation technologies for mitigation^40^. A recent study conducted in various townships reported that phthalic acid esters were detected in all of soil samples (249 samples), with concentrations ranging from 7.3 to 1,064 µg/kg^41^. In addition, 46.8% of agricultural product samples (203 samples) tested positive, with levels varying from below the detection limit to 5,140 µg/kg^41^. Shariati et al.^42^ reported that the mean phthalate concentration was higher in non-agricultural areas compared to agricultural areas. In both types of soil, the highest concentration of phthalates was attributed to bis(2-ethylhexyl) phthalate (59.6%), followed by dioctyl phthalate (27.0%), dimethyl phthalate (11.5%), and diisobutyl phthalate (1.7%)^42^. Currently, studies on phthalate contamination in forage crops are limited. To the best of our knowledge, the origin of bis(2-ethylhexyl) phthalate in *S. grandiflora*, whether it results from environmental contamination or endogenous biosynthesis, has not yet been clarified in the literature. Nevertheless, its detection suggests that the use of SGP as animal feed should be approached with caution, as it may pose ecological risks. For example, feeding SGP could serve as a significant route of phthalate exposure, potentially affecting animal performance and posing risks to human health through the food chain. Kaur et al.^35^ suggested that the half-life of phthalates in soil ranges from 10 to 20 days, with a median lethal dose (LD50) of 1 to 30 g/kg body weight. Due to health concerns, various methods for degrading bis(2-ethylhexyl) phthalate have been explored. However, biodegradation under aerobic or anaerobic conditions using microorganisms, primarily various bacterial strains along with a limited number of fungi, yeasts, and algae, has been suggested to be more effective and sustainable than physical or chemical methods^35^. Similarly, Kong et al.^39^ revealed that bioremediation approaches, such as microbial degradation and phytoremediation, are commonly investigated to address these concerns. In fact, microbial populations present during ensiling and in the rumen are abundant, and their microbiological characteristics, including the use of specific microbial inoculants or direct-fed microbes, may play a significant role in the biodegradation of phthalates in dietary plants. However, this hypothesis requires further investigation.

Forage legumes often present challenges in producing high-quality silage^15^. Given the importance of silage additives, economic feasibility is a key consideration. Based on local market prices and the dosages used in this study, the estimated costs for FA, molasses, and *L. casei* TH14 are approximately 522 (14.93), 500 (14.29), and 400 (11.43) THB (USD), respectively, per 1,000 kg of pre-ensiling material. The results indicate that both FA and MO + TH14 additives significantly improved the ensiling characteristics of SGP silage by modifying (p < 0.001) pH, ethanol, organic acid, and ammonia-N levels compared to the control (Table 4). These findings in the control treatment may be attributed to undesirable levels of WSC and LBC, combined with an insufficient LAB population (Table 1), which contributed to the difficulty of producing high-quality silage from SGP. In contrast, FA treatment induced rapid acidification, inhibiting most microorganisms from the initial stage and significantly reducing ensiling end products such as ethanol and lactic acid. Meanwhile, MO + TH14 exhibited a stimulatory effect, enhancing lactic acid fermentation compared to the control. Unlike direct acid addition, the biological accumulation of lactic acid occurs more gradually, allowing epiphytic microorganisms to produce other acids and ethanol during the initial stage before being inhibited by the resulting acidic conditions. These findings on the effects of ensiling inhibitors and stimulators align with those reported in previous studies^19,43^.

The enhanced proliferation of LAB in the MO + TH14 treatment (Fig. 1) could be attributed to the introduction of the *L. casei* TH14 inoculant, a specific strain of homofermentative LAB that is more competitive and efficient in utilizing WSC substrates than natural microbes^17^. In this study, the synergistic effect of molasses and *L. casei* TH14 may have suppressed the growth of undesirable microorganisms, such as coliforms and yeasts, thereby improving the overall fermentation quality of SGP silage compared to the control. Similarly, Ni et al.^44^ demonstrated that the combination of molasses and LAB effectively inhibited the growth of coliform bacteria, yeasts, and molds in soybean silage. Coliforms and yeasts are considered undesirable in silage due to their involvement in butyric acid production through amino acid decarboxylation^45^. Mold populations were undetectable across all treatments, suggesting that anaerobic conditions were effectively maintained during fermentation. This is beneficial, as mold contamination is a major cause of spoilage and mycotoxin production^46^. However, we found that microbial counts in the FA treatment were absent, except for aerobic bacteria. This dominance of aerobic bacteria in FA-treated silage contradicts previous findings^43,47^. A possible explanation is that the strong acidification induced by FA in this study (7.5 mL/kg FM) rapidly suppressed most microbial populations, including LAB and other fermentative microbes, while creating conditions that favored the survival of acid-tolerant aerobic bacteria. Additionally, variations in silage composition, environmental factors, or differences in microbial communities present in the pre-ensiling material may have contributed to the discrepancy between our findings and previous studies.

Furthermore, our results indicate that FA treatment significantly reduced (p < 0.001) ensiling loss (Fig. 2A) while enhancing aerobic stability (Fig. 2B) compared to other treatments. These findings suggest that silage additives play a key role in influencing the final yield and post-ensiling management efficiency of SGP silage. In the absence of liquid effluents, ensiling losses have been attributed to the production of gases such as carbon dioxide (CO_2_), CH_4_, and nitrous oxide^48^. The inhibitory effect of FA on microbial metabolism likely reduced the microbial-driven production of these gases, leading to lower ensiling loss and improved silage preservation. The FA treatment improved aerobic stability compared to the control (213 vs. 130 h), consistent with a previous study^49^. However, the reduced aerobic stability period (17 h) observed with the MO + TH14 treatment underscores a significant limitation of this additive combination during the feed-out phase. This may be attributed to dormant microorganisms, an abundance of lactic acid as substrate, and insufficient anti-aerobic microbial activity. Chen et al.^50^ noted that homofermentative LAB inoculants may usually reduce aerobic stability due to their preservation of substrates that support the growth of aerobic microorganisms. During the feed-out phase, yeasts and aerobic bacteria are undesirable in silage because they consume lactic acid and other fermentation end-products, leading to an elevated pH and spoilage upon exposure to air^14,51^. However, the concurrent production of anti-aerobic microbial compounds may further support the use of homofermentative LAB inoculants. For instance, Chen et al.^50^ demonstrated that the addition of propionic acid (an effective anti-aerobic microbial compound) at 3.0 g/kg FM, with or without *L. plantarum*, prevented indicators of aerobic spoilage (such as increased pH and the loss of lactic acid and WSC) over 12 days of air exposure, compared to the control or *L. plantarum* alone. Therefore, to further enhance the aerobic stability of SGP silage, future research should consider using complex additive mixtures.

The enhancement of chemical composition can improve the nutritive value of ruminant diets. The results suggest that both FA and MO + TH14 treatments increased (p < 0.05) the DM and OM contents while decreasing (p < 0.05) the NDF and ADF contents of SGP silage compared to the control (Table 5). These findings can be attributed to the enrichment of acidic conditions, which help preserve DM and OM contents^14^ and may also chemically hydrolyze fiber components^52^. Similar findings have been reported in previous studies^43,53,54^. However, in the MO + TH14 treatment, we presume that its chemical composition was also influenced by the amount of molasses added (50 g/kg FM), which potentially provided residual WSC and led to greater ensiling end-product formation than in the other treatments in this study. Wang et al.^19^ also reported that residual WSC increased with higher molasses levels. Additionally, our results indicate that the CP and EE contents of the MO + TH14 treatment did not differ from the control, whereas the FA treatment increased CP content but decreased the EE content of SGP silage. In contrast, in whole-plant mulberry silage, Hao et al.^47^ reported that the addition of glucose + LAB did not affect CP content but improved EE content, while FA did not alter either. The change in CP content observed in this study may be due to the rapid and strong restriction of protein deamination and decarboxylation^16^. Furthermore, this addition of FA may hydrolyze triglycerides, thereby reducing the EE value of SGP silage. Our findings also suggest that FA treatment did not alter the ADL content compared to the control, which aligns with previous reports^43,47^.

The results suggest that both FA and MO + TH14 treatments increased (p < 0.001) IVDMD relative to the control (Table 6). These findings are possibly due to the use of additives, which significantly preserved fermentable nutrients for utilization in the rumen. Zhang et al.^53^ demonstrated that the addition of FA might contribute to a reduction in anti-nutritional factors, such as tannins, thereby increasing DM digestibility and growth rates in goats fed banana pseudostem silage. Dong et al.^18^ reported that the residual WSC in silage prepared with a molasses additive, utilized by rumen microorganisms, resulted in increased digestibility. In addition, Mandebvu et al.^52^ indicated that certain microbial inoculants can enhance fiber digestibility. In this study, these beneficial effects may have also occurred, thereby enhancing IVDMD in SGP silage prepared with additives compared to the control. However, some previous studies have reported that these silage additives did not alter DM digestibility^18,43,50^.

The results also found that the levels of VFAs, GP, and CH_4_ increased (p *<* 0.001) by both silage additives relative to the control (Table 6). Generally, the fermentable nutrients in the rumen are converted into VFAs, microbial cells, and gases such as CO_2_, hydrogen (H_2_), and CH_4_, through diverse metabolic pathways^22^. Therefore, silage additives that effectively enhanced the fermentable nutrients could perform greater rumen fermentation potentials in this study. In addition, the results suggest that the propionic acid/acetic acid ratio in the MO + TH14 treatment, were superior to those in both the FA and control treatments. This is probably due to the greater lactic acid content in MO + TH14-treated silage. Previous studies have been suggested that lactic acid-utilizing bacteria (LUB) in the rumen, such as *Megasphaera elsdenii*, *Selenomonas ruminantium*, and *Propionibacterium* species, can convert lactic acid to propionic acid via the acrylate pathway^55,56^. Another reason is that the residual WSC in MO + TH14-treated silage may further enhance propionic acid production while reducing CH_4_ emissions (g/kg DM) compared to the FA treatment. Hristov et al.^57^ revealed that, under certain conditions, the fermentation of sugars and starches can shift rumen fermentation toward propionic acid production, which usually appears to interfere with methanogenesis.

However, when considering the intensities of in vitro CH_4_ emissions (Fig. 3A and B), the results suggest that emissions expressed as g/kg IVDMD (Fig. 3A) increased with the FA treatment but decreased with the MO + TH14 treatment compared to the control. In our study, while the FA treatment improved IVDMD values similarly to the MO + TH14 treatment (Table 6), the two additives differed in their chemical compositions, particularly in fiber content (Table 5). As a result, they produced SGP silage with varying substrate availability, which may have led to microbial shifts and subsequent effects on methanogenesis in the rumen. A previous study by Zhang et al.^53^ demonstrated that the addition of FA, compared to corn flour in silage, can alter the population of ruminal cellulolytic bacteria. Based on our findings, the relatively higher CH_4_ intensity (g/kg IVDMD) observed in the FA treatment indicates an undesirable effect, limiting its suitability for use in SGP silage production. The effect of FA treatment may be related to the microbial utilization of FA in the rumen^58^ or changes in the properties of bioactive compounds in SGP silage due to acidification^12,53 ^thereby altering CH_4_ inhibitory effects. In general, FA can serve as a substrate for methanogenesis via synthesis pathways^58^. Regarding the effects of MO + TH14 treatment, the consistent reductions in CH_4_ intensities, including those in mol/mol total VFAs (Fig. 3B), are likely attributed to the ruminal LUB’s utilization of lactic acid to produce propionic acid^55,56^. In our study, the MO + TH14 treatment resulted in a significantly higher lactic acid content (Table 4), which may have facilitated an enhanced acrylate pathway compared to the other treatments. This pathway converts lactic acid to lactyl-CoA, leading to the predominant formation of propionic acid while facilitating H_2_ utilization (lactic acid + H_2_ → propionic acid + H_2_O), possibly redirecting H_2_ from hydrogenotrophic methanogenesis (CO_2_ + 4H_2_ → CH_4_ + 2H_2_O)^59^. In a previous study, Cao et al.^61^ suggested that vegetable silage with a high lactic acid content could reduce in vitro CH_4_ production, likely due to electron utilization during propionic acid synthesis. However, the use of silage rich in lactic acid as a feeding strategy to reduce CH_4_ emission intensities may require specific LUB species to achieve effective mitigation, as some species, such as *S. ruminantium*, can utilize the random pathway^59^.

In conclusion, while SGP offers nutritional promise with its CP, true protein, PUFAs, and beneficial phytogenic compounds, including phenolic acids, flavonoids, and antioxidants, its presence of bis(2-ethylhexyl) phthalate content and low IVDMD values warrant cautious application. Furthermore, the suboptimal levels of LBC, WSC, and LAB in the absence of additives hinder the production of high-quality silage. Our findings support the use of MO + TH14 as an effective silage additive to promote lactic acid fermentation, lower pH, improve nutrient availability, and enhance in vitro rumen fermentation, particularly by increasing propionic acid production and reducing CH_4_ intensities (expressed as g/kg IVDMD and mol/mol total VFAs) in SGP silage. These findings underscore the need for in vivo trials to confirm the CH_4_ mitigation potential of SGP silage and evaluate the risk of bis(2-ethylhexyl) phthalate transfer to animal products.

## Methods

The animal experimental protocols conducted in this study were approved by the Institutional Animal Care and Use Committee of Rajamangala University of Technology Isan, with Record No. 35/2565. All animal experiments were complied with the ARRIVE guidelines and relevant laws and were carried out in accordance with the Ethics of Animal Experimentation of the National Research Council, Thailand.

All experimental procedures, including the collection of *S. grandiflora* plant material, were carried out in accordance with relevant institutional, national, and international guidelines and legislation.

## Experimental design, and silage preparation

A completely randomized design with 4 small-scale silo replications was employed to evaluate the effects of silage additives, including control, FA, and MO + TH14. The application rates were selected to ensure appropriate acidification or lactic acid fermentation level, as follows: FA (98% purity) was applied at 7.5 mL/kg FM, molasses (general-grade product) at 50 g/kg FM, and *L. casei* TH14 inoculant at 1.0 × 10^5^ cfu/g FM. *L. casei* TH14 was isolated from corn silage and characterized as a homofermentative lactic acid bacterium^17^.

Fresh, mature, yellow-green pods of *S. grandiflora* were collected in October 2022 from smallholder farmers in Sakon Nakhon Province, Thailand (7.3793° N, 103.7289° E). The pods were harvested from 12 trees, which were approximately 2 to 4 years old, 5 to 9 m tall, and planted without the application of water or fertilizer. The plant material (*S. grandiflora*) used in this study was identified by Dr. Waroon Khota, a specialist in forage crop sciences, based on morphological characteristics and following the descriptions provided by The World Flora Online (https://www.worldfloraonline.org/taxon/wfo-0000178509). A voucher specimen was not deposited in a public herbarium. The pods were pooled and immediately chopped into approximately 1 to 2 cm pieces. Twelve 500 g portions were added with the designated additives. FA or *L. casei* TH14 inoculant was sprayed onto the materials, while molasses was spread over them. Before spraying, *L. casei* TH14 inoculant was mixed with 0.5 mL of normal saline solution.

The pre-ensiling materials were mixed thoroughly with their additive and packed into Hiryu KN type polyethylene bag silos (Asahi Kasei Pax Corp., Tokyo, Japan). Four bags, each containing 400 g, were ensiled for each treatment. They were closed using an SQ-303 vacuum sealer (Asahi Kasei Pax Corp.), stored at room temperature (21 °C to 35 °C), and opened after 30 days. As reported by Pholsen et al.^17 ^a 30-day ensiling period is identified as a stable phase in tropical silage production.

### Ensiling loss, aerobic stability, and microorganism analysis

To measure the ensiling loss, the net weights at before and after ensiling were recorded. The weight loss was calculated and subtracted from the initial net weight, and the result was reported in g/kg.

To measure aerobic stability, the silo was opened. Silage sample (200 g each) was loosely placed into a 500 mL glass beaker, covered with two layers of cheesecloth, and stored in a laboratory at ambient temperature. Temperature readings were frequently taken at the center of the silage using a CyberScan pH/Ion 510 m equipped with a temperature probe (Eutech Instruments Pte Ltd., Singapore). The time (h) on which the silage temperature increased by 1.0 °C above the ambient room temperature was considered as the end of aerobic stability period according to Weiß et al.^62^.

The total plate count technique was used following the method of Kozaki et al.^63^. Fresh SGP and silage samples (10 g each) were immediately mixed with 90 mL of sterilized distilled water, with each preparation performed in triplicate. The mixture was then serially diluted in a sterile normal saline solution from 10^‒1^ to 10^‒5^. Each 20-µL sample of the diluted mixture was spread on prepared agar plates. Lactobacilli de Man, Rogosa, Sharpe agar (Difco Laboratories Inc., Detroit, MI, USA) was prepared for counting LAB, blue light broth agar (Nissui Ltd., Tokyo, Japan) for coliform bacteria, nutrient agar (Difco Laboratories Inc.) for aerobic bacteria, and potato dextrose agar (Nissui Ltd.) for counting yeasts and molds. LAB were counted after incubation at 30 °C for 48 h in an anaerobic chamber (Sugiyamagen Ltd., Tokyo, Japan). Coliform bacteria were counted after incubation at 30 °C for 48 h, while aerobic bacteria, yeasts, and molds were counted after incubation at 30 °C for 24 h. Colony appearance and cell morphology were used to differentiate between yeasts and molds. The limit of detection was < 10^2^ cfu/g FM, and the results were expressed as log10 cfu/g FM.

### pH, LBC, ethanol, organic acids, and ammonia-N analysis

The levels of pH, LBC, ethanol, organic acids, and ammonia-N were measured from cold water extracts following the method outlined by Cai et al.^64^. Sample (10 g each) was mixed with 90 mL of sterilized distilled water and incubated in refrigerator for overnight, with each preparation performed in triplicate.

Following incubation, the mixture was brought to 25 °C, and pH measurement was conducted using a FiveGo pH meter (Mettler-Toledo GmbH, Greifensee, Switzerland). The mixture of fresh SGP sample was then analyzed for LBC using the titration technique as described by McDonald et al.^15^. The pH was adjusted from its initial value to pH 3.00 using 0.1 normal hydrochloric acid solution, followed by continuous stirring for 5 min. Subsequently, the sample underwent gradual titration with 0.1 normal sodium hydroxide (NaOH) solution to elevate the pH value. The volume of 0.1 normal NaOH solution required to shift the pH from 4.00 to 6.00 was measured and used to calculate the LBC value, expressed in mEq/kg DM.

The concentrations of ethanol and organic acids in the mixture were measured with a periodic acid reagent method, modified from the procedure described by Darwin et al.^65^. The sample was added with 2-methylvaleric acid as the internal standard. The analysis was conducted using a GC-2010 Plus GC (Shimadzu Co., Kyoto, Japan) equipped with a DB-WAX capillary column (30 m, 0.25 mm, 0.25 μm, Agilent Technologies, Inc., Santa Clara, CA, USA) and a flame ionization detector (FID). Helium was used as carrier and makeup gas. The GC-FID conditions were set as follows: 0.4 µL injection volume (1:10 split ratio, 250 °C temperature, 29.1 cm/sec linear velocity, and 3 mL/min purge flow), gradient column temperatures (50 °C hold 2 min, 75 °C/min to 130 °C hold 10 min, and 75 °C/min to 200 °C hold 1 min), and 280 °C FID temperature (30 mL/min makeup gas, 30 mL/min H_2_, and 400 mL/min air). The total program time was 15 min. Three linear concentrations of standard mixtures were used for quantification. The ammonia-N content of silage sample was measured using a UV/VIS spectrophotometer (PG Instruments Ltd., London, UK) following the Nessler’s reagent method described by Fawcett and Scott^66^. All results were reported in g/kg DM.

### Chemical composition analysis

The DM content of SGP material and silage samples was analyzed at 100 °C for 24 h using an UF450 oven dryer (Memmert GmbH, Schwabach, Germany). Also, the samples (100 g each) were dried at 60 °C for 48 h and then ground to pass through a 1 mm sieve screen using an IKA MF10 grinding mill (IKA Werke GmbH & Co. KG, Staufen, Germany). The standard methods outlined in AOAC^67^ were employed to analyze the OM (method 942.05) and EE (method 920.39) contents. The CP content was analyzed using an 828 Series: Elemental Analysis by Combustion N analyzer (LECO, St. Joseph, MI, USA) based on 6.25 factor of total N content. The NDF and ADF contents were analyzed using their respective detergents as described by Van Soest et al.^68^ with an ANKOM 200 fiber analyzer (ANKOM Technology, Macedon, NY, USA). For NDF analysis, α-amylase was included. The ADL was determined by solubilization with a 72%v/v sulfuric acid solution^69^. The NDF, ADF, and ADL contents were expressed inclusive of ash residue. The WSC content of the SGP material were analyzed using the spectrophotometer (PG Instruments Ltd.), employing the Dubois’s phenol-sulfuric acid method described by Kerepesi et al.^70^. The protein fractions of the SGP material, including soluble true protein, soluble non-true protein, and insoluble protein, were analyzed using the bicarbonate-phosphate buffer method described by Krishnamoorthy et al.^23^. The soluble true protein content was measured using the spectrophotometer (PG Instruments Ltd.) based on the Lowry protein assay^23 ^while the insoluble protein content was determined with the N analyzer (LECO). N values were converted to protein by multiplying by 6.25. The soluble non-true protein content was calculated as the difference (soluble non-true protein = CP − soluble true protein − insoluble protein). All results were expressed as g/kg DM.

### In vitro rumen fermentation analysis

An in vitro rumen fermentation method^71^ was used to evaluate IVDMD, GP, individual VFAs, and CH_4_ values after 24 h of incubation. Approximately 0.50 g of milled dry SGP material and silage samples were weighed into pre-weighed F57 fiber analysis bags (ANKOM Technology). Three bags per sample were prepared along with three blanks. The blanks contained empty fiber analysis bags. The bags were heat-sealed and placed into 50 mL serum bottles. All bottles were then sealed with a rubber stopper and an aluminum seal cap and incubated at 39 °C in an SI600 orbital shaker incubator (Stuart, Staffordshire, UK). Buffer solution^71^ was prepared and warmed to 39 °C with CO_2_ gas flushing.

The beef cattle used in this experiment were obtained from the Beef Cattle Research and Development Unit, Faculty of Natural Resources, Rajamangala University of Technology Isan, Sakon Nakhon Campus, Sakon Nakhon, Thailand. Adequate rumen fluid (approximately 200 mL/head) was collected using a stomach tube before morning feeding from 2 healthy, mature male Zebu×Angus (75:25) crossbred beef cattle. The average body weight of cattle was 300 ± 20 kg. In daytime, the cattle were allowed to graze on the grassland. In nighttime, they were housed with their herd with a free choice of rice straw, mineral block and drinking water. The rumen fluid with low saliva contamination was immediately pooled, transferred to the laboratory, filtered through 4 layers of cheesecloth, mixed with the buffer solution (1:4), and flushed with CO_2_ gas. A 40 mL portion of the mixed rumen fluid medium was injected into each bottle. All bottles were incubated at 39 °C while shaking at 60 rpm (SI600).

The method for measuring GP and CH_4_ was outlined in our previous work^20^. Briefly, GP was measured and stored at intervals of 2, 4, 6, 8, 10, 12, 16, 20, and 24 h using a calibrated glass syringe. The CH_4_ concentration was analyzed using the GC-2010 Plus equipped with an SH-Rt-Q-BOND capillary column (30 m, 0.53 mm, 20 μm, Shimadzu Co.). Helium was used as carrier and makeup gas. The GC-FID conditions were set as follows: 10 µL injection volume (1:10 split ratio, 250 °C temperature, 31 cm/sec linear velocity, and 3 mL/min purge flow), 150 °C isothermal column temperature, and 250 °C FID temperature (30 mL/min makeup gas, 40 mL/min H_2_, and 400 mL/min air). The analysis end time was set at 2.0 min. Four levels of high-purity CH_4_ (5, 10, 15, and 20%v/v in N_2_ gas) were served as the standard. The volume of CH_4_ emissions was converted using 22.414 L/mol and 16.04 g/mol, respectively. The residual samples were washed with pepsin solution and distilled water, dried at 100 °C in the air-drying oven for 24 h, and weighed to determine IVDMD in g/kg.

The concentration of the individual VFAs in the in vitro rumen fluid was analyzed using a diethyl ether extraction method^72 ^with 2-methylvaleric acid as the internal standard. The analysis employed the same GC system used for the analysis of ethanol and organic acids. The GC-FID conditions were set as follows: 1 µL injection volume (1:10 split ratio, 250 °C temperature, 35.6 cm/sec linear velocity, and 3 mL/min purge flow), gradient column temperatures (120 °C hold 2.5 min, 8 °C/min to 140 °C, and 12 °C/min to 190 °C hold 0.83 min), and 270 °C FID temperature (24 mL/min makeup gas, 32 mL/min H_2_, and 200 mL /min air). The total program time was 10 min. The results were reported in mol/kg DM substrate.

### Fatty acid profile analysis

The fatty acid profile of SGP material was analyzed using a direct fatty acid methyl ester (FAME) synthesis method, modified from the procedure described by O’Fallon et al.^73^. Milled dry SGP material (0.50 g) was added with 0.7 mL of 10 normal potassium hydroxide in water and 6.3 mL of methanol. The reaction mixture was incubated horizontally at 55 °C while shaking at 120 rpm for 90 min (SI600), then cooled in cold water. Subsequently, 0.58 mL of sulfuric acid was added slowly. The tubes were then re-incubated horizontally and cooled in cold water again for an additional round. Hexane (3 mL) was added, and the mixture was gently vortexed and centrifuged to obtain the hexane layer. Finally, 1.5 mL of the hexane layer was transferred to a GC vial and stored at ‒20 °C for the FAMEs analysis.

The GC system was similar to those used for ethanol and organic acid analysis. The GC-FID conditions were set as follows: 1.0 µL injection volume (1:50 split ratio, 250 °C temperature, 30.1 cm/sec linear velocity, and 3 mL/min purge flow), gradient column temperatures (50 °C hold 1 min, 25 °C/min to 200 °C, and 3 °C/min to 230 °C hold 21 min), and 280 °C FID temperature (30 mL/min makeup gas, 40 mL/min H_2_, and 400 mL/min air). The total program time was 38 min. A Food Industry FAME Mix (Cat. No. 35077) containing 37 compounds (30 mg total/mL in methylene chloride; Restek Co., Benner Circle, Bellefonte, PA, USA) served as the standard. Prior to use, 0.5 mL of the standard was mixed with 1 mL of hexane. Only the identified target peaks were taken into the account and reported as g/kg of total fatty acids (FAMEs Eq).

### Phytochemical composition, antioxidant activity, and GC-MS analysis

Methanol extraction using a Soxhlet apparatus (10 h) and a rotary evaporator was performed to obtain crude extracts from milled dry SGP material (3 replicates), following the method described by Iqbal et al.^74^. The crude extracts were dehydrated at 60 °C for 48 h, weighed (g/kg DM), and pooled before being used in the analyses.

As outlined by Iqbal et al.^74 ^total phenolics, total flavonoids, and antioxidant activity were spectrophotometrically measured using an Infinite 200 PRO microplate reader (Tecan Trading AG, Männedorf, Switzerland). The Folin–Ciocalteu reagent with gallic acid as the standard was used to determine the content of total phenolics. The content of total flavonoids was measured using the aluminum reagent, with quercetin as the standard. Antioxidant activity, or free radical scavenging capacity, was assessed using the 2,2-diphenyl-1-picrylhydrazyl (DPPH) assay, with trolox as the standard. The results were reported in mg Eq of standard per g of extract. Following the reagent tests as described in previous studies^74,75 ^the crude extract was screened for alkaloid (Wagner’s test), anthraquinone (Borntrager’s test), cardiac glycoside (Keller-Kiliani’s test), coumarin (NaOH test), saponin (frothing test), tannin (ferric chloride test), and terpenoid (Salkowski’s test). The results were reported as either positive (+) or negative (‒) test.

GC-MS analysis was conducted following the method reported by our previous work^76^. The crude extract was dissolved in methanol to achieve a concentration of 0.2 mg/mL. The analytical setup utilized the GC-2010 Plus equipped with an SH-Rtx-5MS capillary column (30 m, 0.32 mm, 0.25 μm, Shimadzu Co.) and a GCMS-QP2020 detector (Shimadzu Co.). Helium was used as carrier gas and operated under an electron impact ionization of 70 eV. The GC-MS conditions were set as follows: 1.0 µL injection volume (1:50 split ratio, 250 °C temperature, 42.5 cm/sec linear velocity, and 3 mL/min purge flow), gradient column temperatures (50 °C, 5 °C/min to 140 °C hold 5 min, 3 °C/min to 200 °C, and 15 °C/min to 260 °C), 280 °C MS temperature (2 min solvent cut time, relative to the tuning result of detector gain mode, 1.09 kV detector gain, and 200 threshold), and MS Table (2.5 min start time, 47 min end time, 1,428 scan speed, 35 start m/z, and 400 end m/z). Chemical constituents were identified by comparing their relative retention indices with a NIST17 library.

### Statistical analysis

The data obtained from SGP silage were analyzed using a one-way analysis of variance (ANOVA) of SAS Version 6.12^77^. The statistical model is as follows:

Y_ij_ = µ + τ_i_ + ε_ij_.

Where Y_ij_ = observation; µ = overall mean, τ_i_ = silage additive effect, and ε_ij_ = error. The treatment mean differences were determined by Duncan’s new multiple range test at p *<* 0.05 following the method of Steel and Torrie^78^.

## Data Availability

All data supporting the findings of this study are available within the paper.

## References

[1] IPCC. Refinement to the 2006 IPCC guidelines for national greenhouse gas inventories volume 4 agriculture, forestry and other land use. In *Intergovernmental Panel on Climate Change (IPCC)* (2019). https://www.ipcc-nggip.iges.or.jp/public/2019rf/vol4.html (2019).

[2] Gerber, P. J. et al. *Tackling Climate Change Through Livestock–A Global Assessment of Emissions and Mitigation Opportunities* (Food and Agriculture Organization of the United Nations, 2013).

[3] Khejornsart, P., Cherdthong, A. & Wanapat, M. In vitro screening of plant materials to reduce ruminal protozoal population and mitigate ammonia and methane emissions. *Fermentation* **7**, 166 (2021).

[4] Nastoh, N. A., Waqas, M., Çınar, A. A. & Salman, M. The impact of phytogenic feed additives on ruminant production: A review. *J. Anim. Feed Sci.* **33**, 431–453 (2024).

[5] Janardhana, K. et al. Experimental investigation on utilization of *Sesbania grandiflora* residues through thermochemical conversion process for the production of value added chemicals and biofuels. *Sci. Rep.* **14**, 7283 (2024). 38538627 10.1038/s41598-024-57040-y PMC10973372

[6] Unnawong, N., Cherdthong, A. & So, S. Crude saponin extract from *Sesbania grandiflora* (L.) pers pod meal could modulate ruminal fermentation, and protein utilization, as well as mitigate methane production. *Trop. Anim. Health Prod.* **53**, 196 (2021). 33674897 10.1007/s11250-021-02644-z

[7] Aye, M. M., Aung, H. T., Sein, M. M. & Armijos, C. A review on the phytochemistry, medicinal properties and pharmacological activities of 15 selected Myanmar medicinal plants. *Molecules* **24**, 293 (2019). 30650546 10.3390/molecules24020293 PMC6359042

[8] Rani, D. M. et al. Anti-cancer bioprospecting on medicinal plants from Indonesia: A review. *Phytochemistry* **216**, 113881 (2023). 37827225 10.1016/j.phytochem.2023.113881

[9] Ash, A. J. The effect of supplementation with leaves from the leguminous trees *Sesbania grandiflora*, *Albizia chinensis* and *Gliricidia sepium* on the intake and digestibility of guinea grass hay by goats. *Anim. Feed Sci. Technol.* **28**, 225–232 (1990).

[10] Unnawong, N., Cherdthong, A. & So, S. Influence of supplementing *Sesbania grandiflora* pod meal at two dietary crude protein levels on feed intake, fermentation characteristics, and methane mitigation in thai purebred beef cattle. *Vet. Sci.* **8**, 35 (2021). 33672399 10.3390/vetsci8020035 PMC7926297

[11] Rajappan, A., Smitha, R. & Krishnan, P. Phytochemical and antibacterial studies on seeds of *Sesbania grandiflora* Linn.: A novel report. *J. Pharmacogn. Phytochem*. **13**, 142–153 (2024).

[12] Ngwa, A. T., Nsahlai, I. V. & Bonsi, M. L. K. Feed intake and dietary preferences of sheep and goats offered hay and legume-tree pods in South Africa. *Agrofor. Syst.* **57**, 29–37 (2003).

[13] Kung, L., Shaver, R. D., Grant, R. J. & Schmidt, R. J. *Silage review:* Interpretation of chemical, microbial, and organoleptic components of silages. *J. Dairy Sci.* **101**, 4020–4033 (2018). 29685275 10.3168/jds.2017-13909

[14] Muck, R. E. et al. *Silage review:* Recent advances and future uses of silage additives. *J. Dairy Sci.* **101**, 3980–4000 (2018). 29685273 10.3168/jds.2017-13839

[15] McDonald, P., Henderson, A. & Heron, S. *The Biochemistry of Silage* (Chalcombe, 1991).

[16] Wei, S. N., Li, Y. F., Jeong, E. C., Kim, H. J. & Kim, J. G. Effects of formic acid and lactic acid bacteria inoculant on main summer crop silages in Korea. *J. Anim. Sci. Technol.* **63**, 91–103 (2021). 33987587 10.5187/jast.2021.e7 PMC7882833

[17] Pholsen, S., Khota, W., Pang, H., Higgs, D. & Cai, Y. Characterization and application of lactic acid bacteria for tropical silage preparation. *Anim. Sci. J.* **87**, 1202–1211 (2016). 26799939 10.1111/asj.12534

[18] Dong, Z., Wang, S., Zhao, J., Li, J. & Shao, T. Effects of additives on the fermentation quality, in vitro digestibility and aerobic stability of mulberry (*Morus alba* L.) leaves silage. *Asian-Australas. J. Anim. Sci.* **33**, 1292–1300 (2020). 32054226 10.5713/ajas.19.0420 PMC7322647

[19] Wang, J. et al. Effects of molasses on the fermentation characteristics of mixed silage prepared with rice straw, local vegetable by-products and alfalfa in Southeast China. *J. Integr. Agric.* **16**, 664–670 (2017).

[20] Kaewpila, C. et al. Improving ensiling characteristics by adding lactic acid bacteria modifies in vitro digestibility and methane production of forage-sorghum mixture silage. *Sci. Rep.* **11**, 1968 (2021). 33479407 10.1038/s41598-021-81505-z PMC7820244

[21] NRC. *Nutrient Requirements of Beef Cattle* (National Academy, 2000).

[22] Van Soest, P. J. *Nutritional Ecology of the Ruminant* (Cornell University Press, 1994).

[23] Krishnamoorthy, U., Muscato, T. V., Sniffen, C. J. & Van Soest, P. J. Nitrogen fractions in selected feedstuffs. *J. Dairy Sci.* **65**, 217–225 (1982).

[24] Ortiz-Domínguez, G. et al. Nutritional value and in vitro digestibility of legume pods from seven trees species present in the tropical deciduous forest. *Trop. Subtrop. Agroecosystems*. **20**, 505–510 (2017).

[25] Ngwa, T. A., Nsahlai, I. V. & Iji, P. A. Ensilage as a means of reducing the concentration of cyanogenic glycosides in the pods of *Acacia sieberiana* and the effect of additives on silage quality. *J. Sci. Food Agric.* **84**, 521–529 (2004).

[26] Ndlovu, L. R. Complementarity of forages in ruminant digestion: Theoretical consideration. In *The Complementarity of Feed Resources for Animal Production* (eds. Stares, J. E. S., Said, A. N. & Kategile, J. A.). 18–21 ILCA (1992).

[27] Thiputen, S. & Sommart, K. Prediction equations of metabolisable energy content in beef cattle diets. *Khon Kaen Univ. Res. J.* **17**, 35–44 (2012).

[28] Palmquist, D. Institute of Food and Agricultural Sciences, University of Florida, Gainesville. Essential fatty acids in ruminant diets. In *21st Annual Ruminant Nutrition Symposium*. 127–141 (2010).

[29] Golbotteh, M. M., Malecky, M., Aliarabi, H. & Zamani, P. Impact of oil type and savory plant on nutrient digestibility and rumen fermentation, milk yield, and milk fatty acid profile in dairy cows. *Sci. Rep.* **14**, 22427 (2024). 39341950 10.1038/s41598-024-73138-9 PMC11438970

[30] Alves, S. P., Mendonça, S. H., Silva, J. L. & Bessa, R. J. B. *Nannochloropsis oceanica*, a novel natural source of rumen-protected eicosapentaenoic acid (EPA) for ruminants. *Sci. Rep.* **8**, 10269 (2018). 10.1038/s41598-018-28576-7 PMC6035222 29980726

[31] Zain, M., Despal, T. U. H., Pazla, R., Putri, E. M. & Amanah, U. Evaluation of legumes, roughages, and concentrates based on chemical composition, rumen degradable and undegradable proteins by in vitro method. *Int. J. Vet. Sci.* **12**, 528–538 (2023).

[32] Deepthi, K., Renjith, P. K., Shameem, K., Habeeb Rahman, K. & Chandramohanakumar, N. Phytochemical screening of leaves and flower extracts of *Sesbania grandiflora* (L.) pers. and its antimicrobial activity against fish pathogens. *Vegetos* **36**, 626–633 (2023).

[33] Namysl, S. et al. A first evaluation of butanoic and pentanoic acid oxidation kinetics. *Chem. Eng. J.* **373**, 973–984 (2019).

[34] Priyanto, J. A. et al. Bioactivity potential and chemical profile of endophytic *Stutzerimonas stutzeri* strain D2 isolated from *Myristica fatua* houtt. *J. Res. Pharm.* **28**, 51–62 (2024).

[35] Kaur, R., Kumari, A., Rajput, V. D., Minkina, T. & Kaur, R. Biodegradation of phthalates and metabolic pathways: an overview. *Environ. Sustain.* **6**, 303–318 (2023).

[36] Shafikova, T. N., Maksimova, L. A., Omelichkina, Y. V., Enikeev, A. G. & Semenov, A. A. Endogenous phthalates in plants and their alleged participation in defense response against phytopathogens. *IOP Conf. Ser. Earth Environ. Sci.* **408**, 012076 (2020).

[37] Shariati, S., Pourbabaei, A. A., Alikhani, H. A., Rezaei, K. A. & Shariati, F. Phthalic acid esters as pervasive emerging pollutants in the environment and their role in threatening food security and human health: A review. *Iran. J. Soil Water Res.* **52**, 2253–2277 (2021).

[38] Sahnsarayi, S. K., Shariati, F. & Karimzadegan, H. The pollution load of phthalates in the effluent of plastic recycling units in the coastal areas of the Southern Caspian Sea. *Water Air Soil Pollut*. **236**, 94 (2025). 10.1007/s10661-025-14102-6 40455109

[39] Kong, X. et al. Pollution status, ecological effects, and bioremediation strategies of phthalic acid esters in agricultural ecosystems: A review. *J. Agric. Food Chem.* **72**, 27668–27678 (2024). 39620367 10.1021/acs.jafc.4c07884

[40] Das, M. T. et al. Remediation strategies for mitigation of phthalate pollution: Challenges and future perspectives. *J. Hazard. Mater.* **409**, 124496 (2021). 33187797 10.1016/j.jhazmat.2020.124496

[41] Li, H. et al. Distribution features and health risk assessment of phthalate pollutants in facility soil and agricultural products in Xinjiang, China. *Agronomy* **15**, 821 (2025).

[42] Shariati, F., Gaskaminjan, S. N., Galangash, M. M. & Ooshaksaraei, L. Phthalate contamination in agricultural and non-agricultural soils around landfills in Western and Central Gilan Province. *SSRN* 19 (2024). 10.2139/ssrn.4829202

[43] Zhao, J. et al. Effect of storage time and the level of formic acid on fermentation characteristics, epiphytic microflora, carbohydrate components and in vitro digestibility of rice straw silage. *Anim. Biosci.* **34**, 1038–1048 (2021). 33906266 10.5713/ajas.20.0388 PMC8100481

[44] Ni, K. et al. Effects of lactic acid bacteria and molasses additives on the microbial community and fermentation quality of soybean silage. *Bioresour. Technol.* **238**, 706–715 (2017). 28501002 10.1016/j.biortech.2017.04.055

[45] Ma, J. et al. Silage additives improve fermentation quality, aerobic stability and rumen degradation in mixed silage composed of amaranth and corn straw. *Front. Plant. Sci.* **14**, 1189747 (2023). 37426969 10.3389/fpls.2023.1189747 PMC10325724

[46] Penagos-Tabares, F. et al. Fungal species and mycotoxins in mouldy spots of grass and maize silages in Austria. *Mycotoxin Res.* **38**, 117–136 (2022). 35347677 10.1007/s12550-022-00453-3 PMC9038934

[47] Hao, L. et al. Formic acid enhances whole-plant mulberry silage fermentation by boosting lactic acid production and inhibiting harmful bacteria. *Front. Microbiol.* **15**, 1399907 (2024). 38915298 10.3389/fmicb.2024.1399907 PMC11194324

[48] Tian, J., Tian, R., Wu, J., Huang, L. & Zhang, J. Gas production characteristics of oats and tritical silages and techniques for reducing gas emissions. *J. Integr. Agric.* **24**, 1246–1258 (2025).

[49] Hao, J. et al. Fermentation quality, bacterial community, and aerobic stability of perennial recut *Broussonetia Papyrifera* silage with different additives and wilting time. *Fermentation* **8**, 262 (2022).

[50] Chen, L. et al. Effect of lactic acid bacteria and propionic acid on conservation characteristics, aerobic stability and in vitro gas production kinetics and digestibility of whole-crop corn based total mixed ration silage. *J. Integr. Agric.* **16**, 1592–1600 (2017).

[51] Ran, Q. et al. Effect of formic acid and inoculants on microbial community and fermentation profile of wilted or un-wilted italian ryegrass silages during ensiling and aerobic exposure. *Fermentation* **8**, 755 (2022).

[52] Mandebvu, P. et al. Effect of enzyme or microbial treatment of bermudagrass forages before ensiling on cell wall composition, end products of silage fermentation and in situ digestion kinetics. *Anim. Feed Sci. Technol.* **77**, 317–329 (1999).

[53] Zhang, H., Cheng, X., Elsabagh, M., Lin, B. & Wang, H. Effects of formic acid and corn flour supplementation of banana pseudostem silages on nutritional quality of silage, growth, digestion, rumen fermentation and cellulolytic bacterial community of Nubian black goats. *J. Integr. Agric.* **20**, 2214–2226 (2021).

[54] Huo, W. et al. Effect of lactic acid bacteria on the ensiling characteristics and in vitro ruminal fermentation parameters of alfalfa silage. *Ital. J. Anim. Sci.* **20**, 623–631 (2021).

[55] McAllister, T. A. et al. Review: The use of direct fed microbials to mitigate pathogens and enhance production in cattle. *Can. J. Anim. Sci.* **91**, 193–211 (2011).

[56] McAllister, T. A. & Newbold, C. J. Redirecting rumen fermentation to reduce methanogenesis. *Aust. J. Exp. Agric.* **48**, 7–13 (2008).

[57] Hristov, A. N. et al. SPECIAL TOPICS—Mitigation of methane and nitrous oxide emissions from animal operations: I. A review of enteric methane mitigation options. *J. Anim. Sci.* **91**, 5045–5069 (2013). 24045497 10.2527/jas.2013-6583

[58] Sun, K., Liu, H., Fan, H., Liu, T. & Zheng, C. Research progress on the application of feed additives in ruminal methane emission reduction: A review. *PeerJ* **9**, e11151 (2021). 10.7717/peerj.11151 PMC8019312 33850664

[59] Ushida, K., Hoshi, S. & Ajisaka, K. ^13^C-NMR studies on lactate metabolism in a porcine gut microbial ecosystem. *Microb. Ecol. Health Dis.* **14**, 242–247 (2009).

[60] Chen, L. et al. *Megasphaera elsdenii* lactate degradation pattern shifts in rumen acidosis models. *Front. Microbiol.* **10**, 162 (2019). 30792704 10.3389/fmicb.2019.00162 PMC6374331

[61] Cao, Y. et al. Effect of lactic acid bacteria inoculant and beet pulp addition on fermentation characteristics and in vitro ruminal digestion of vegetable residue silage. *J. Dairy Sci.* **94**, 3902–3912 (2011). 21787927 10.3168/jds.2010-3623

[62] Weiß, K., Kroschewski, B. & Auerbach, H. U. The influence of delayed sealing and repeated air ingress during the storage of maize silage on fermentation patterns, yeast development and aerobic stability. *Fermentation* **8**, 48 (2022).

[63] Kozaki, M., Uchimura, T. & Okada, S. *Experimental Manual for Lactic Acid Bacteria* (Asakurasyoten, 1992).

[64] Cai, Y., Benno, Y., Ogawa, M. & Kumai, S. Effect of applying lactic acid bacteria isolated from forage crops on fermentation characteristics and aerobic deterioration of silage. *J. Dairy Sci.* **82**, 520–526 (1999). 10194670 10.3168/jds.S0022-0302(99)75263-X

[65] Darwin, Charles, W. & Cord-Ruwisch, R. Concurrent lactic and volatile fatty acid analysis of microbial fermentation samples by gas chromatography with heat pre-treatment. *J. Chromatogr. Sci.* **56**, 1–5 (2018). 29069353 10.1093/chromsci/bmx086

[66] Fawcett, J. K. & Scott, J. E. A rapid and precise method for the determination of urea. *J. Clin. Pathol.* **13**, 156–159 (1960). 13821779 10.1136/jcp.13.2.156 PMC480024

[67] AOAC. *Official Methods of Analysis* (Association of Official Analytical Chemists, 1990).

[68] Van Soest, P. J., Robertson, J. B. & Lewis, B. A. Methods for dietary fiber, neutral detergent fiber, and nonstarch polysaccharides in relation to animal nutrition. *J. Dairy Sci.* **74**, 3583–3597 (1991). 1660498 10.3168/jds.S0022-0302(91)78551-2

[69] Galyean, M. L. *Laboratory Procedures in Animal Nutrition Research* (Department of Animal and Food Science, Texas Tech University, 1997).

[70] Kerepesi, I., Tóth, M. & Boross, L. Water-soluble carbohydrates in dried plant. *J. Agric. Food Chem.* **44**, 3235–3239 (1996).

[71] Makkar, H. P., Blümmel, M. & Becker, K. Formation of complexes between polyvinyl pyrrolidones or polyethylene glycols and tannins, and their implication in gas production and true digestibility in in vitro techniques. *Br. J. Nutr.* **73**, 897–913 (1995). 7632671 10.1079/bjn19950095

[72] Chuntrakort, P. et al. The effect of dietary coconut kernels, whole cottonseeds and sunflower seeds on the intake, digestibility and enteric methane emissions of zebu beef cattle fed rice straw based diets. *Livest. Sci.* **161**, 80–89 (2014).

[73] O’Fallon, J. V., Busboom, J. R., Nelson, M. L. & Gaskins, C. T. A direct method for fatty acid methyl ester synthesis: application to wet meat tissues, oils, and feedstuffs. *J. Anim. Sci.* **85**, 1511–1521 (2007). 17296772 10.2527/jas.2006-491

[74] Iqbal, E., Salim, K. A. & Lim, L. B. L. Phytochemical screening, total phenolics and antioxidant activities of bark and leaf extracts of *Goniothalamus velutinus* (Airy Shaw) from Brunei Darussalam. *J. King Saud Univ. - Sci.* **27**, 224–232 (2015).

[75] Kumar, R. S., Venkateshwar, C., Samuel, G. & Rao, S. G. Phytochemical screening of some compounds from plant leaf extracts of *Holoptelea integrifolia* (Planch.) and *Celestrus emarginata* (Grah.) used by Gondu tribes at Adilabad District, Andhrapradesh, India. *Int. J. Eng. Sci. Invent.* **2**, 65–70 (2013).

[76] Tongkasee, P. et al. Formulation and phytochemical profile of a product prototype infused with *Cannabis sativa* leaves. *Trends Sci.* **20**, 5835 (2023).

[77] SAS. *SAS User’s Guide: Statistic 6.12* (SAS Institute Inc., 1996).

[78] Steel, R. G. D. & Torrie, J. H. *Principles and Procedures of Statistics: A Biometrical Approach* (McGraw–Hill Book Co. Inc., 1980).

